# A case report of laparoscopic surgery for Mayer-Rokitansky-Küster-Hauser syndrome with preservation of functional primordial uterus

**DOI:** 10.1186/s12905-023-02741-1

**Published:** 2023-11-27

**Authors:** Wei Zhao, Naiyi Du, Luguang Han, Yakun Liu, Ying Wang, Xiwa Zhao, Jun Zhang, Shan Kang

**Affiliations:** Department of Gynecology, the Fourth Hospital of Hebei Medical University, No.12 Health Road, Chang’ an District, Shijiazhuang, 050011 Hebei China

**Keywords:** Laparoscopic surgery, MRKH syndrome, Preservation of functional primordial uterus, Reproductive potential

## Abstract

**Background:**

In the past, the primary treatment for MRKH syndrome (Mayer-Rokitansky-Küster-Hauser syndrome) with a functional primordial uterus was surgical removal of the functional primordial uterus. In rare instances, the endometrium of the functional primordial uterus is well developed, and surgical preservation of the functional primordial uterus provides the possibility of preserving reproductive function for these patients.

**Case presentation:**

A 14-year-old female was diagnosed with type I MRKH syndrome with a functional primordial uterus through physical examination and imaging investigations. We freed the functional primordial uterus through laparoscopic surgery and excised a portion of the lower myometrium to create an outlet at a lower uterine segment, which we then intermittently anastomosed to the tip of the artificial vagina. The patient recovered well after the surgery, and a re-examination showed no significant abnormalities.

**Conclusion:**

We were successful in preserving the functional primordial uterus using laparoscopic surgery in a patient with MRKH syndrome and connecting it to an artificial vagina through reconstructive surgery to ensure unobstructed menstrual drainage and preserve the reproductive potential of the patient.

**Supplementary Information:**

The online version contains supplementary material available at 10.1186/s12905-023-02741-1.

## Background

MRKH syndrome (Mayer-Rokitansky-Küster-Hauser syndrome) is a rare reproductive tract malformation in females caused by embryonic bilateral paramesonephric duct hypoplasia or bilateral paramesonephric duct tail dysplasia, and patients generally seek medical consultation due to primary amenorrhea in adolescence or dyspareunia after marriage [1]. Common clinical anatomical features include bilateral solid primordial uterine nodes with complete deficiency of the vagina or deficiency of the upper 2/3 vagina, cavernous lower 1/3 vagina, and blind tip. The bilateral ovaries and fallopian tubes are normally developed, and some patients may have concomitant urinary, skeletal, cardiac, and other systemic malformations [2].

A small number of patients have a functional primordial uterus where the primordial uterus has a developed endometrium. In this condition, patients often experience recurrent abdominal pain, and the basic treatment in the past consisted of surgical removal of the functional primordial uterus. In a very small number of patients, the functional primordial uterus has a well-developed endometrium and a large uterine cavity, and since the patients have normal ovaries and fallopian tubes, surgical preservation of the functional primordial uterus allows for the preservation of reproductive function in these patients.

A patient with MRKH syndrome with a functional primordial uterus was recently admitted to our hospital, and we performed laparoscopic surgery to preserve the functional primordial uterus on the left side and performed uterovaginal reconstruction with positive results. We present details of the case in the following sections.

## Case presentation

### General information

The patient was a 14-year-old female who was admitted to our hospital mainly due to “periodic lower abdominal pain for one year”. The patient was delivered normally at full term, and her mother had no history of exposure to drugs, toxic substances during pregnancy, or radioactive substances. The patient started developing breasts at the age of 12 and had not had a menstrual cycle to date. One year ago, she started to have lower abdominal pain, which was recurrent (around every 28–30 days) and relieved spontaneously after about a week. At this time, she visited another hospital and underwent a magnetic resonance imaging (MRI) examination, which showed a “bilateral primordial uterus, with the left side being larger (containing a little endometrium); an undeveloped cervix and middle and upper vaginal segments; a left ovarian cyst, with chocolate cyst unable to be excluded”.

Findings of the physical examination on admission: abdominal examination showed no significant abnormality, and no obvious mass was palpated. The gynecological examination showed a young female vulva with normal development and a small amount of pubes. The hymen was normal, and the swab could enter about 2 cm from the hymen. The cervix and uterus were not palpated on rectal abdominal examination. A cystic solid mass with a diameter of about 5 cm was palpated in the left adnexal region with pain on mild pressing, while no obvious mass was palpated in the right adnexal region.

**Fig. 1:**
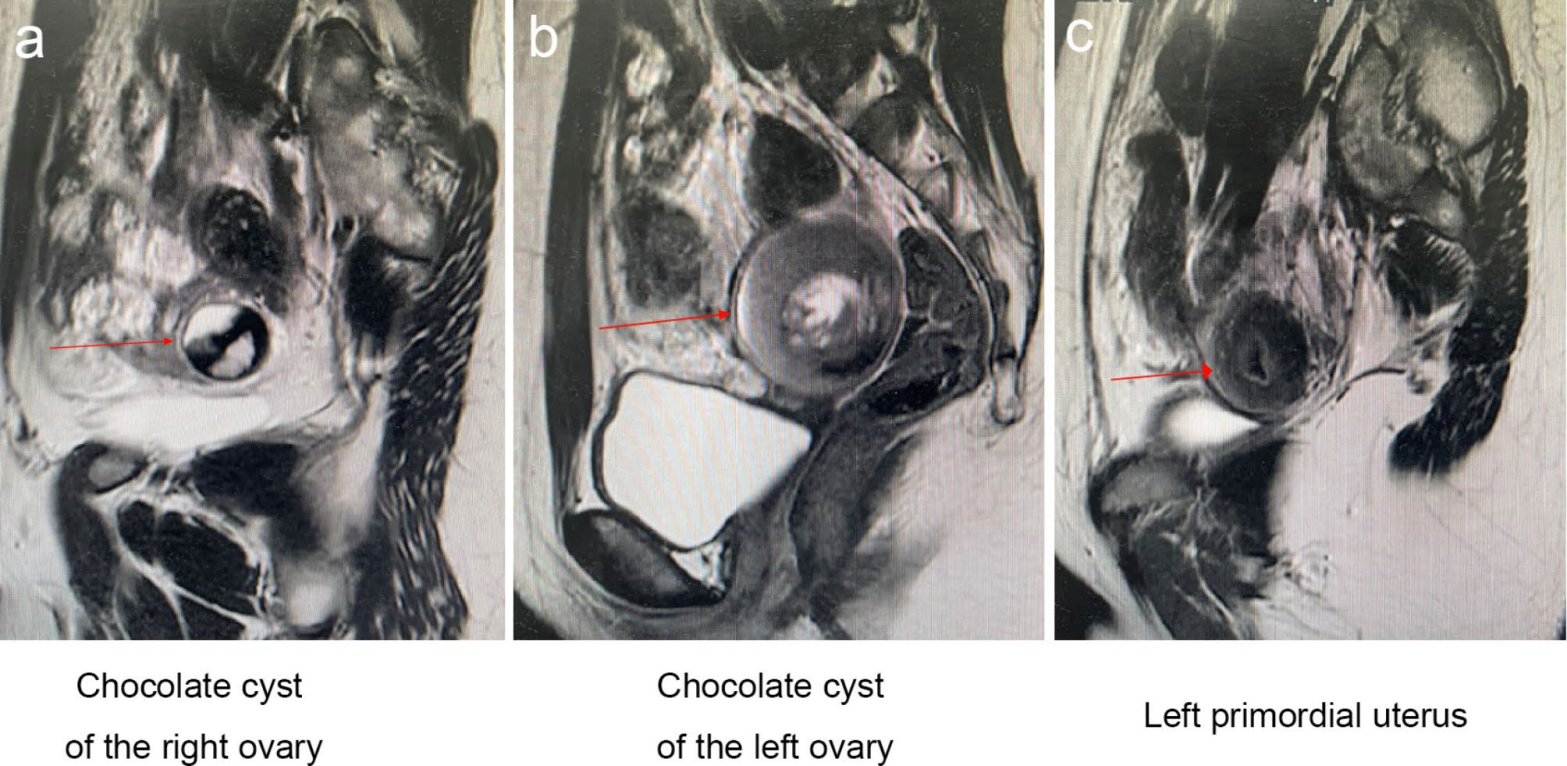
Preoperative MR sagittal view: (a) chocolate cyst of the right ovary; (b) chocolate cyst of the left ovary; (c) left primordial uterus

**Fig. 2:**
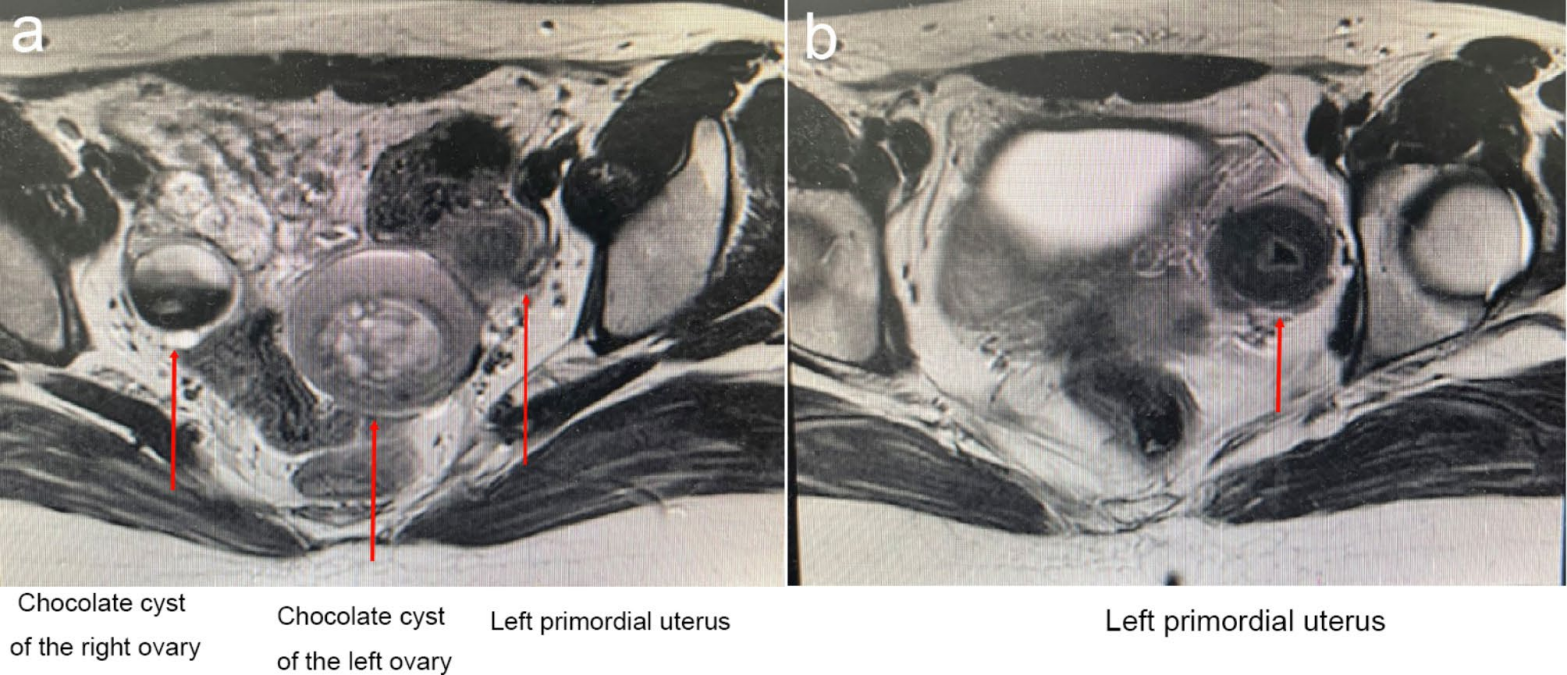
Preoperative MR axial view: (a) chocolate cyst of the right ovary, chocolate cyst of the left ovary, and the left primordial uterus; (b) left primordial uterus

MR scanning (Figs. 1 and 2) suggested a left pelvic node with a mixed shadow of short T1 and long T2 signals, with a length of about 5 cm and a high signal in the diffusion-weighted imaging (DWI). A uterus-like mass was seen on the upper left side of the node, with a length of about 3 cm and an irregular endometrium-like structure inside. The right adnexal region showed a quasi-circular mixed signal node with high signal on the internal DWI sequence and a length of about 2.8 cm. The cervix and the upper vaginal segment were not clearly shown, while the lower vaginal segment was seen with a slit-like vaginal cavity. The diagnostic imaging indicated a primordial uterus on the upper left side of the pelvic cavity, chocolate cyst on the left side of the pelvic cavity, chocolate cyst in the right adnexal region, and an undeveloped cervix and upper vaginal segment. The karyotpe was XX, 46 of the patient. There were no abnormities in heart, renal, vertebral and other organs [2, 3]. Preoperative preliminary diagnosis: (1) type I MRKH syndrome with functional primordial uterus; (2) bilateral ovarian cysts.

## Methods

**Fig. 3:**
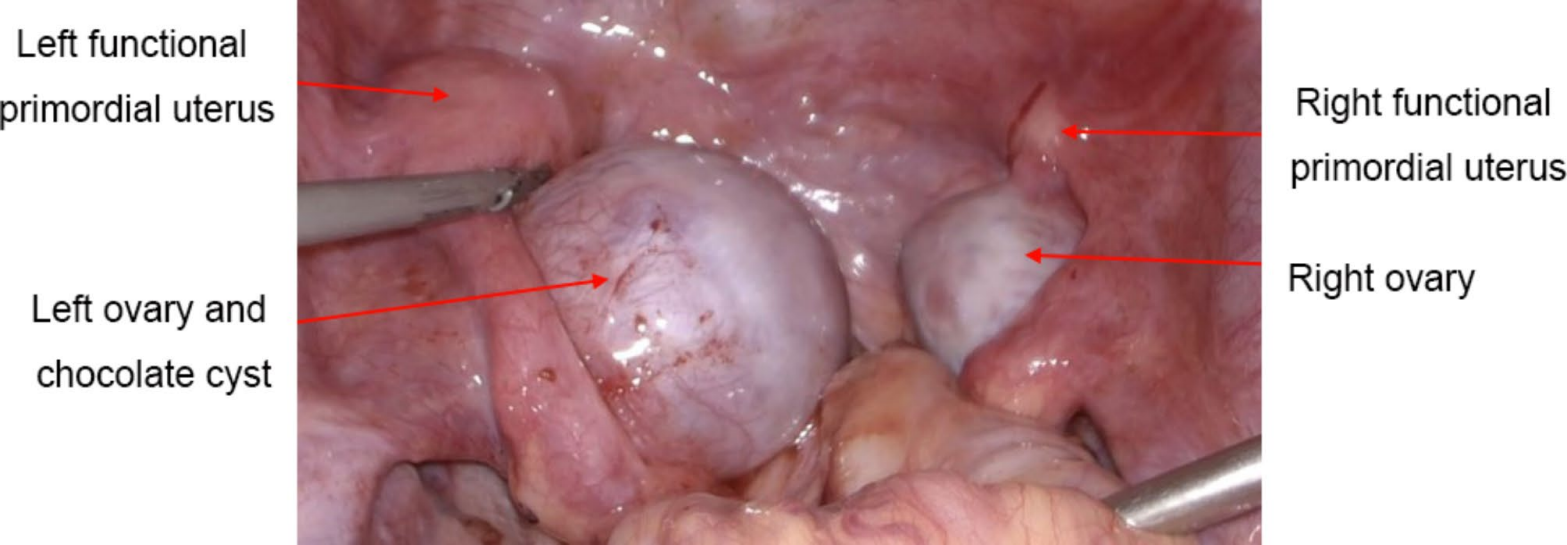
Laparoscopic view of the pelvic cavity

The bowel preparation was done one day before surgery, and the patient was instructed to remain fast without food or water on the day of surgery. The procedure was done with the patient placed in the lithotomy position under general anesthesia. Laparoscopic exploration of the pelvic and abdominal cavities (Fig. 3) showed a small amount of blood in the pelvic cavity, a primordial uterus of about 2 cm × 1 cm × 1 cm on the right pelvic wall, and cystic enlargement of the right ovary with a diameter of about 3 cm. An enlarged primordial uterus of about 5 cm × 4 cm × 4 cm on the left pelvic wall and a cystically enlarged left ovary with a diameter of about 5 cm could be seen immediately to the right of the primordial uterus, and no abnormality was seen in the bilateral fallopian tubes. No obvious ectopic lesions were seen on the peritoneal surface of the pelvic and abdominal cavities.

A bilateral cholecystectomy was performed first, during which chocolate-like fluid outflow was seen from the left ovary (Supplementary Fig. 1) and blood outflow was seen from the right ovary. After the operation, the bilateral ovaries were sutured and shaped. The left pelvic funnel ligament was freed, and the left round ligament and the anterior and posterior lobes of the broad ligament were severed. The left uterine artery and ureter were further freed, the superior branch of the left uterine artery was severed.The fibrous cord tissues of the lower uterine segment, which did not develop into the cervix, were excised (Supplementary Fig. 2).The left parametrium was freed downward to free the left functional primordial uterus as far down as possible in preparation for subsequent anastomosis (Supplementary Fig. 3).

Under laparoscopic monitoring, a cavity was bluntly punched inward along the external vaginal orifice in the middle of the vulvar vestibule, parallel to the urethra and rectum, until the pelvic floor peritoneum in the interstitial space between the bladder and rectum, while saline was injected intermittently to form a water cushion. Oval forceps were placed in the cavity to open the pelvic floor peritoneum, which was incised to create an artificial vaginal tunnel that could accommodate two fingers. The peritoneum on the posterior wall of the bladder was freed completely, with an area of about 6 cm × 6 cm. The freed peritoneum was attached to the mold surface, and a drainage tube was placed through the opening at the top of the mold. The mold was placed into the artificial vaginal tunnel while carrying the peritoneum and drainage tube (Supplementary Fig. 4).

The distal peritoneum was intermittently sutured and anastomosed to the “natural vaginal mucosa” of the lower vaginal segment, and the proximal peritoneum was sutured and fixed to the tip of the artificial vaginal tunnel (Supplementary Fig. 5). The uterine cavity was exposed by a full incision of the freed left functional primordial uterus near the vagina, and the endometrial tissues could be seen inside the uterine cavity without significant blood accumulation (Supplementary Fig. 6). A portion of the myometrium tissues, approximately two cm in diameter, were removed, and the intima at the defective area was intermittently evaginated and sutured to the serous layer so that it would be like a “bowl” and serve as the outlet of the lower uterine segment (Supplementary Fig. 7). Finally, absorbable sutures were used to intermittently anastomose the artificial outlet of the lower uterine segment and the tip of the artificial vagina (Supplementary Fig. 8).

### Operation outcome

The operation was successful, with an operation duration of 2 h and an intraoperative bleeding amount of 30 ml. The patient was administered antibiotics to prevent infection, the vaginal mold was replaced every other day, iodophor solution was used to disinfect and irrigate the vagina, and the vaginal drainage tube was removed two days after the surgery. The ovarian cysts were confirmed as endometriosis cysts by post-operative pathological examination. The patient recovered well post-surgery without any complications and was successfully discharged with the mold six days after the operation.

During the follow-up period, the patient had a menstrual cycle in the first month post-surgery, which lasted for about three days with minimal bleeding, unblocked discharge of menstrual blood, and no abdominal pain. Subsequently, the patient had regular menstrual cycles every month without abdominal pain. The pelvic MR did not show any abnormal mass six months after the surgery, and the functional primordial uterus was connected to the artificial vagina. The postoperative outcome was good.

## Discussion

In 1961, the Swiss scholar Hauser systematically described the pathogenesis and clinical manifestations of MRKH syndrome, summarizing previous reports [4]. For a long time afterwards, especially in China, clinicians have referred to this condition as the “congenital absence of uterus and vagina”. With a rise in the number of cases and a growing interest in studying the disease, more and more scholars have found that some patients with MRKH syndrome do not suffer a complete absence of the “uterus and vagina”; they just do not have normal “uterus and vagina” due to the dysplasia of “uterus and vagina”. They may have a primordial uterus or a lower 1/3 vagina. A retrospective cohort study conducted from 1997 to 2011 at the University of Michigan (USA) in 2013 [5] consisting of 48 patients with MRKH syndrome found that 48% (23 cases) had a primordial uterus. In a review in 2020 [2], it was estimated that 48–84% of patients with MRKH syndrome had a primordial uterus.

Most patients with MRKH syndrome have no clinically significant symptoms as the primordial uterus does not have a functional endometrium. However, if the primordial uterus has a functional endometrium, also known as a functional primordial uterus, clinical symptoms such as abdominal pain are more likely to occur, and patients are more likely to consult a clinician and get diagnosed earlier. In the retrospective cohort study conducted by the University of Michigan (USA) in 2013 [5], MRI confirmed that 19% (9 cases) of 48 patients with MRKH syndrome had a functional primordial uterus, and all of these patients experienced abdominal pain.

Clinicians mainly chose surgical removal of the functional primordial uterus followed by vaginal reconstruction, for patients with MRKH syndrome with a functional primordial uterus associated with severe abdominal pain. This surgery is relatively simple and effective in relieving abdominal pain, but the patient will permanently lose menstruation and reproductive function post-surgery, and this can have a significant psychological impact on the patient. It is for this reason that there are efforts to preserve the functional primordial uterus and reconstruct it with an artificial vagina through surgery so that menstrual blood can flow out smoothly through the artificial vagina, thus preserving the physiological function and potential reproductive function.

As early as 1938, a successful treatment of cervical atresia through uterovaginal reconstruction was reported [6]. Zarou et al. [7] reported the first successful pregnancy following such uterovaginal reconstruction in 1973, demonstrating the feasibility of such surgery. In 2003, Selvaggi et al. [8] successfully treated two cases of MRKH syndrome with a functional primordial uterus using open surgery. The surgeons used the pudendal thigh fasciocutaneous flap to reconstruct the artificial vagina and then completed the anastomosis between the artificial vagina and the functional primordial uterus by open surgery. After the surgery, both patients resumed regular menstruation with unobstructed flow of menstrual blood.

In 2008, Raudrant et al. [9] successfully performed laparoscopic preservation of the functional primordial uterus and uterovaginal reconstruction in a patient with MRKH syndrome. The patient had spontaneous menstrual flow three months after the operation and maintained unobstructed menstruation during the two-year follow-up. In 2018, Professor Luo Guangnan reported the first case in China of laparoscopic preservation of a unilateral functional primordial uterus in a patient with MRKH syndrome [4]. In 2021, Scibilia and collegues reported a case of congenital isolated cervical agenesis receiving laparoscopic uterovaginal anastomosis [10]. They successfully treated a patient with MRKH syndrome with a left functional primordial uterus by the Luohu III laparoscopic peritoneal vaginoplasty procedure (Luohu II Procedure + functional primordial hysterotomy + anastomosis of artificial vaginal and uterus), and the patient had a menstrual cycle one month after the operation. The surgery unblocked the drainage of the uterine cavity and preserved the fertility of the patient.

In our study, the patient had a left ovarian endometriosis cyst, so we first performed the left oophorocystectomy and then freed the left functional primordial uterus as much as possible for subsequent downward traction and anastomosis with artificial vaginal, which was one of the key steps of the procedure. We chose the surface peritoneum of the posterior bladder wall for the peritoneal vaginoplasty as it has a sufficient area of peritoneum and is easy to obtain. In previous uterovaginal reconstruction procedures, a small hole was mainly made at the lower end of the uterus to serve as an outflow tract, and a drainage tube was placed into the uterine cavity through this hole, followed by anastomosis of artificial vaginal and primordial uterus.

However, in our case, the size of the primordial uterus was relatively large, so we creatively excised part of the myometrium at the lower end of the uterus, which was about 2 cm in diameter (equivalent to the diameter of the artificial vagina), until the uterine cavity was fully exposed, and then we intermittently evaginated the endometrium and sutured it to the serous layer to make a “bowl”, which was equivalent to creating a relatively wide outlet at the lower uterine segment; this was another key step of the surgery. Then, the artificial outlet at the lower uterine segment was intermittently anastomosed with the tip of the artificial vagina.

Such a surgery has two advantages. First, the short-term advantage: it eliminates the need for prolonged use of a “uterine drainage tube” post-surgery and requires just a routine change of the vaginal mold, which makes short-term postoperative care easier and can be assigned to the patient’s family. Second, the long-term advantage: the outlet at the lower uterine segment is relatively wide, and this reduces the possibility of adhesion or even obstruction of the outlet at the lower uterine segment due to postoperative inflammation and scarring and avoids reoperation.

The smooth postoperative recovery of the patient also confirmed the advantages of the surgery; the vaginal drainage tube was removed two days after the surgery, and the patient was discharged with the mold six days after the procedure. In the first month post-surgery, the patient had a menstrual cycle with unblocked discharge of menstrual blood and no abdominal pain. Afterwards, the patient had regular menstrual cycles every month without abdominal pain. There was no significant abnormality on re-examination six months after the surgery; no abnormal mass was seen on pelvic MR, and the functional primordial uterus was connected to the artificial vagina.

## Conclusion

We treated this case entirely by laparoscopic operation with minimal surgical trauma, and the patient recovered quickly after the operation. Reconstructive surgery enabled the preservation of the functional primordial uterus and its connection with the artificial vagina, ensuring unblocked menstrual drainage and relieving the stress of the patient and her family. In addition, the fertility potential of the patient was also preserved. The long-term impact and reproductive function are yet to be observed through follow-up.

### Electronic supplementary material

Below is the link to the electronic supplementary material.

Supplementary Material 1

## Data Availability

The data that support the findings of this study are available from the corresponding author, upon reasonable request.
