# Supplementary material for: A case report of laparoscopic surgery for Mayer-Rokitansky-Küster-Hauser syndrome with preservation of functional primordial uterus
Source: BMC Womens Health. 2023 Nov 27;23:634. doi: 10.1186/s12905-023-02741-1 (PMC10683278; doi:10.1186/s12905-023-02741-1)
Supplement: Supplementary file 1 — Supplementary Material 1 [file 12905_2023_2741_MOESM1_ESM.doc]

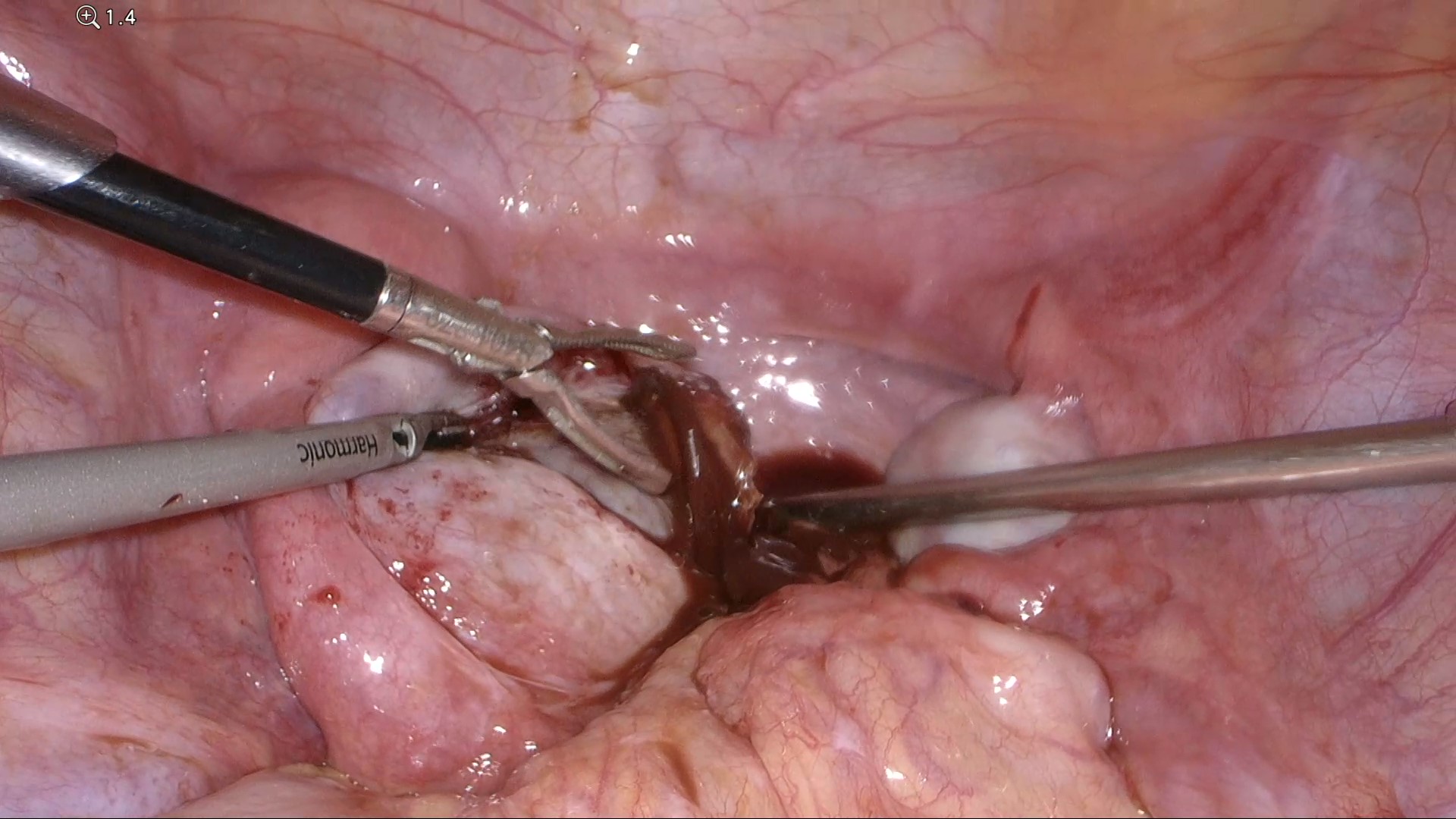


Supplementary figure 1 Chocolate cyst of left ovary


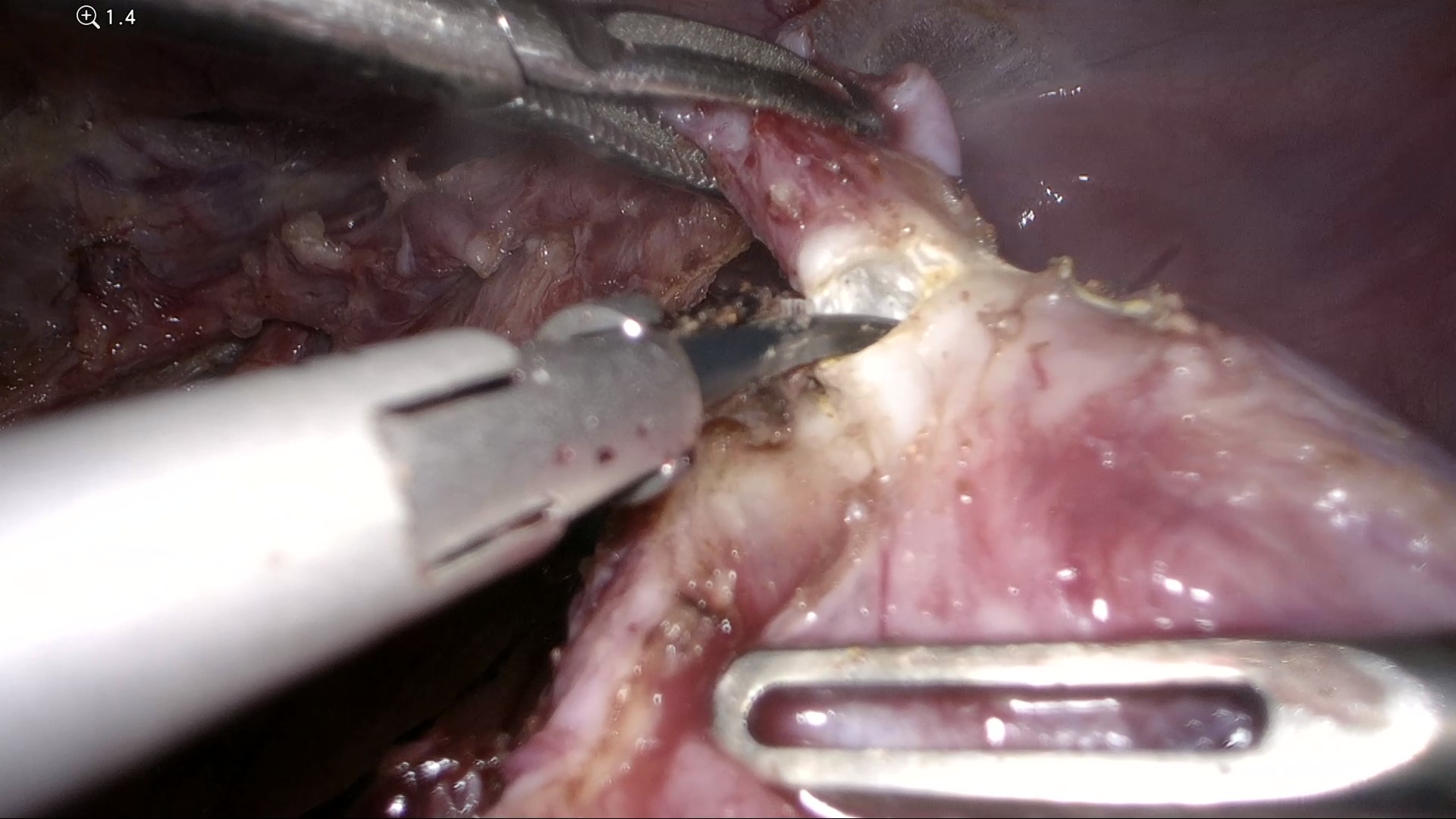


Supplementary figure 2 Cutting the fibrous cord tissue (immature cervix) of the lower uterus.


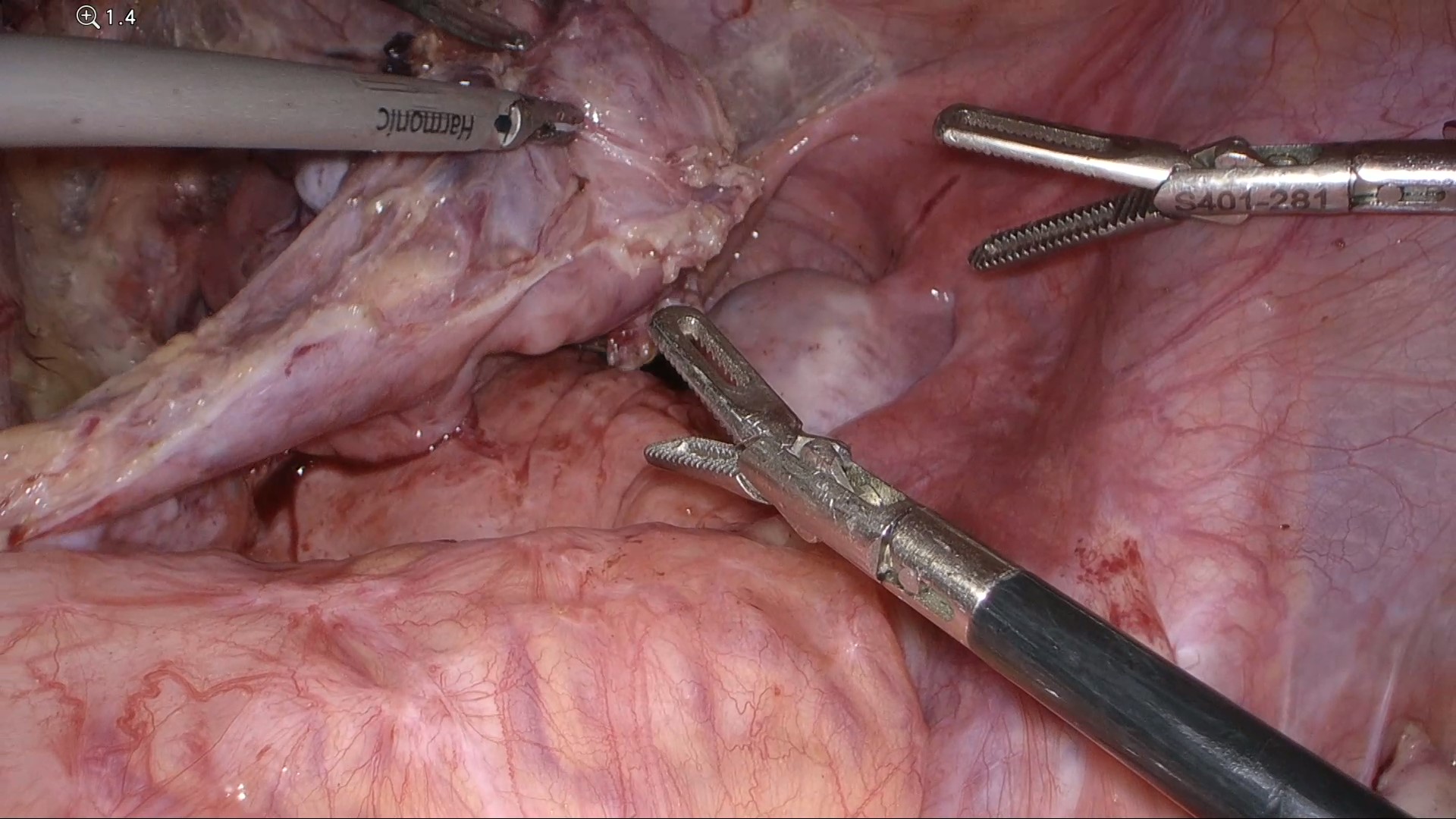


Supplementary figure 3 Pulling down the free left functional primordial uterus for preparation of subsequent anastomosis


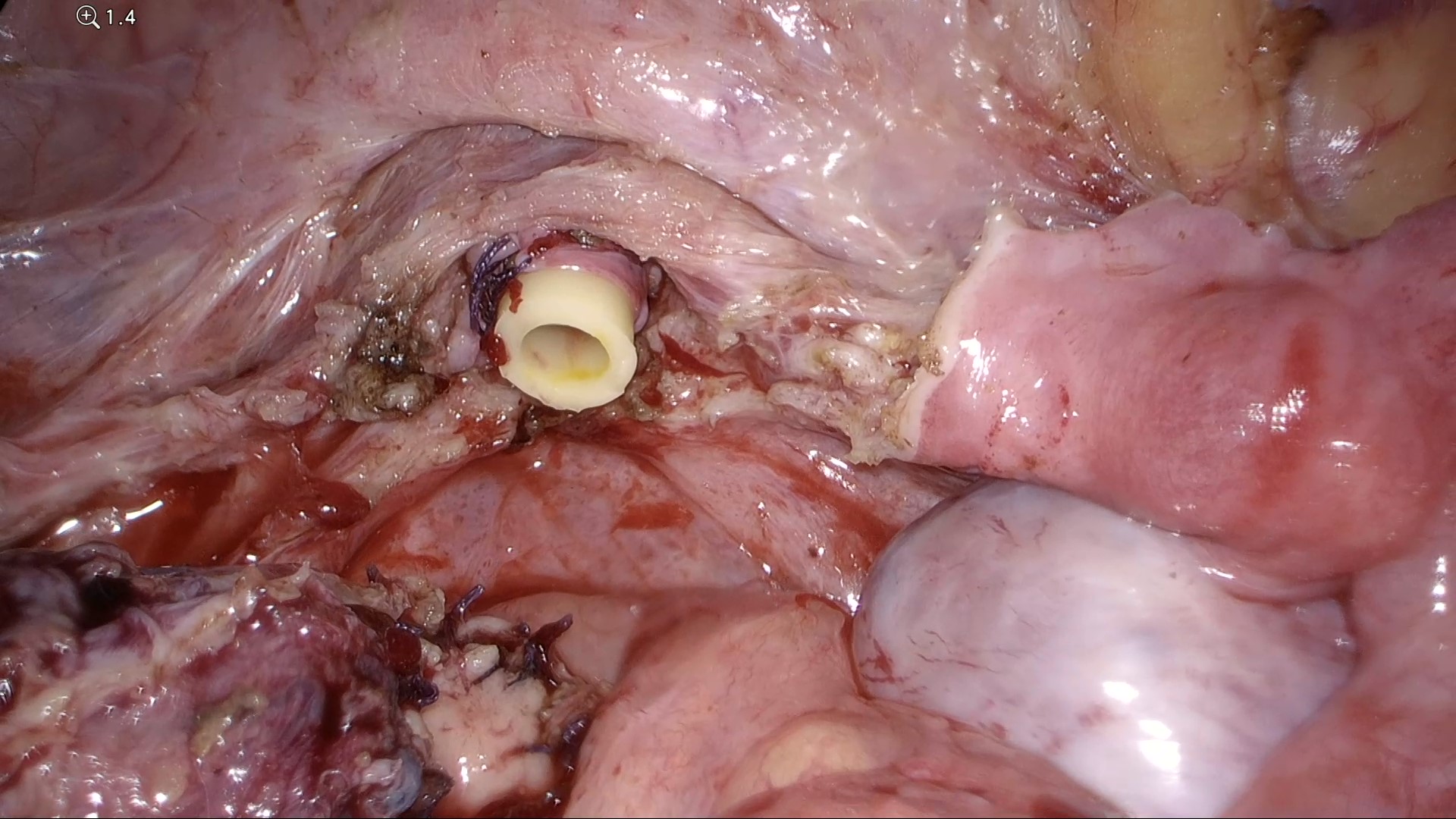


Supplementary figure 4 Implanting the drainage into the artificial vaginal tunnel through the mold


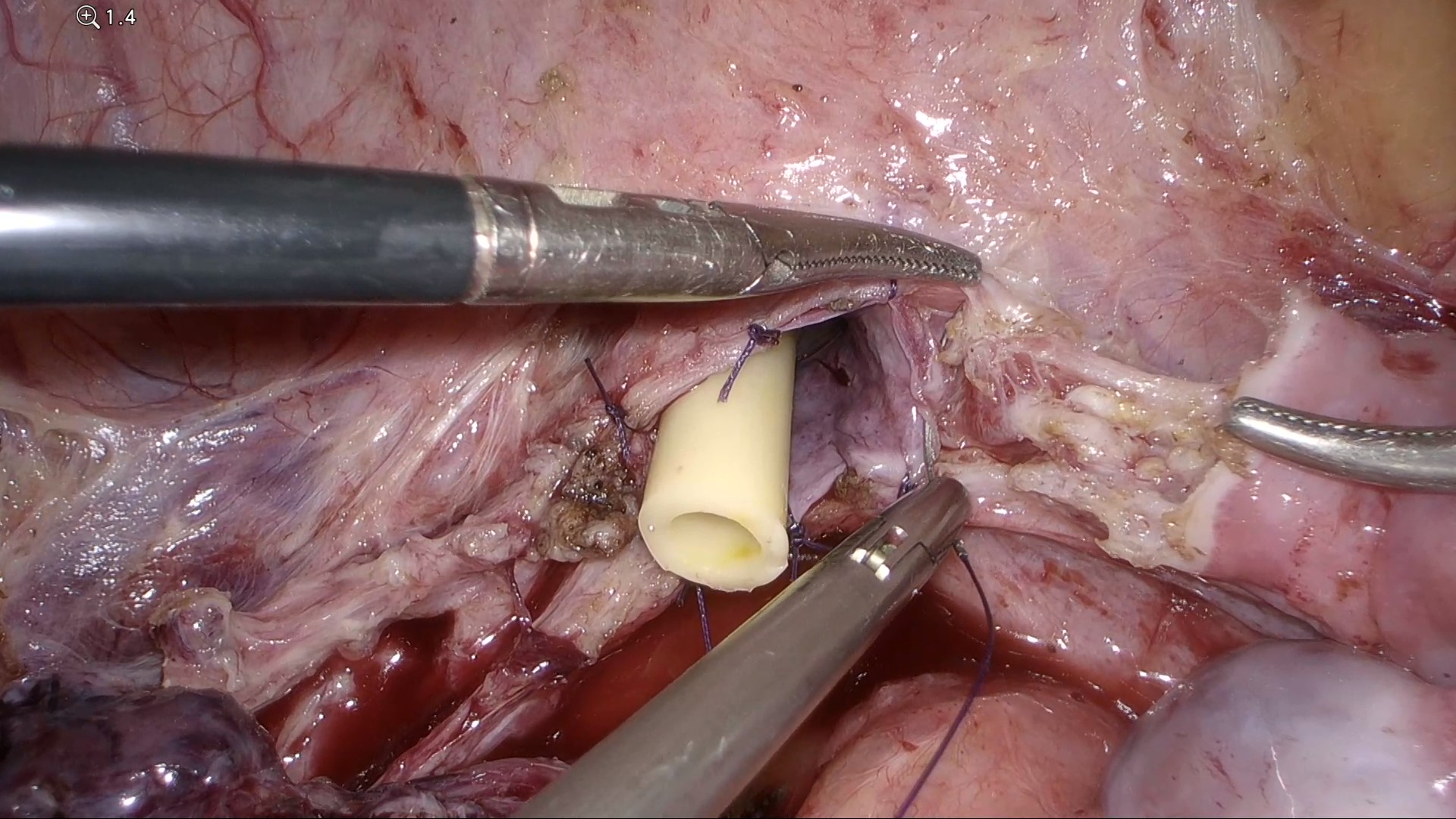


Supplementary figure 5 Peritoneal suture was fixed at the top of the artificial vaginal tunnel as vagina alteration


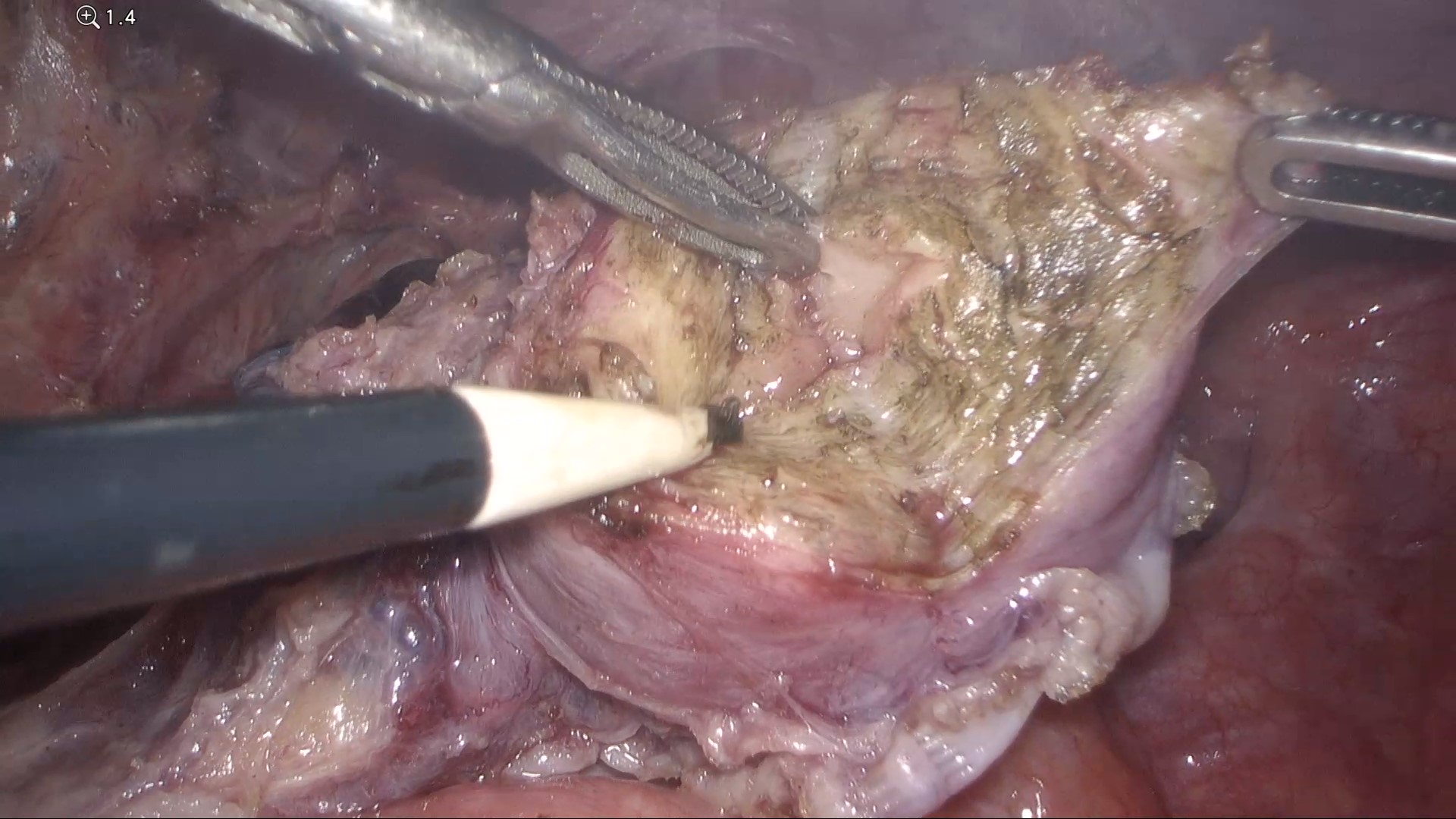


Supplementary figure 6 Incision of the basal myometrium and exposure of the uterine cavity


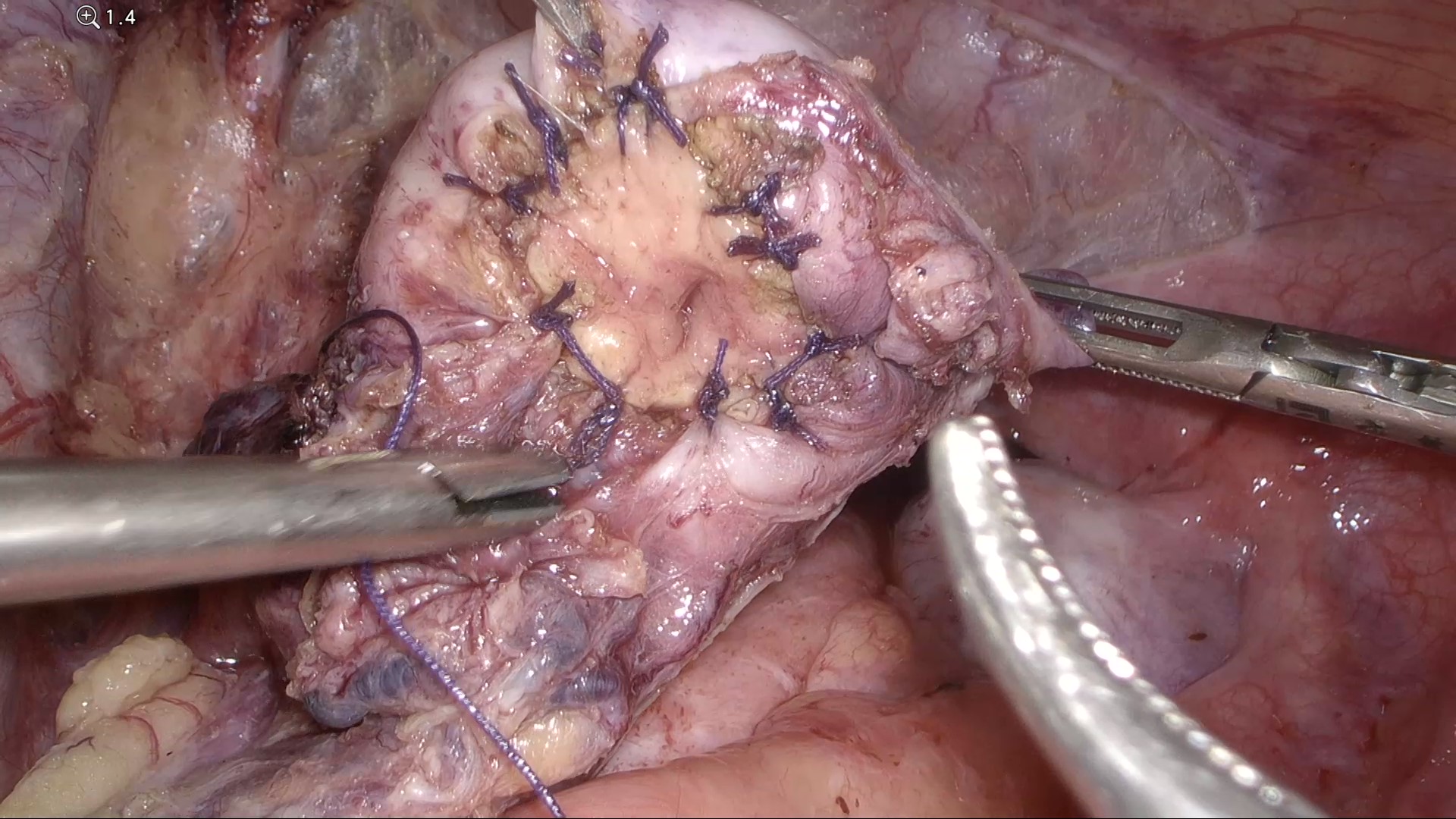


Supplementary figure 7 Artificial "bowl-shape" exit of the uterus


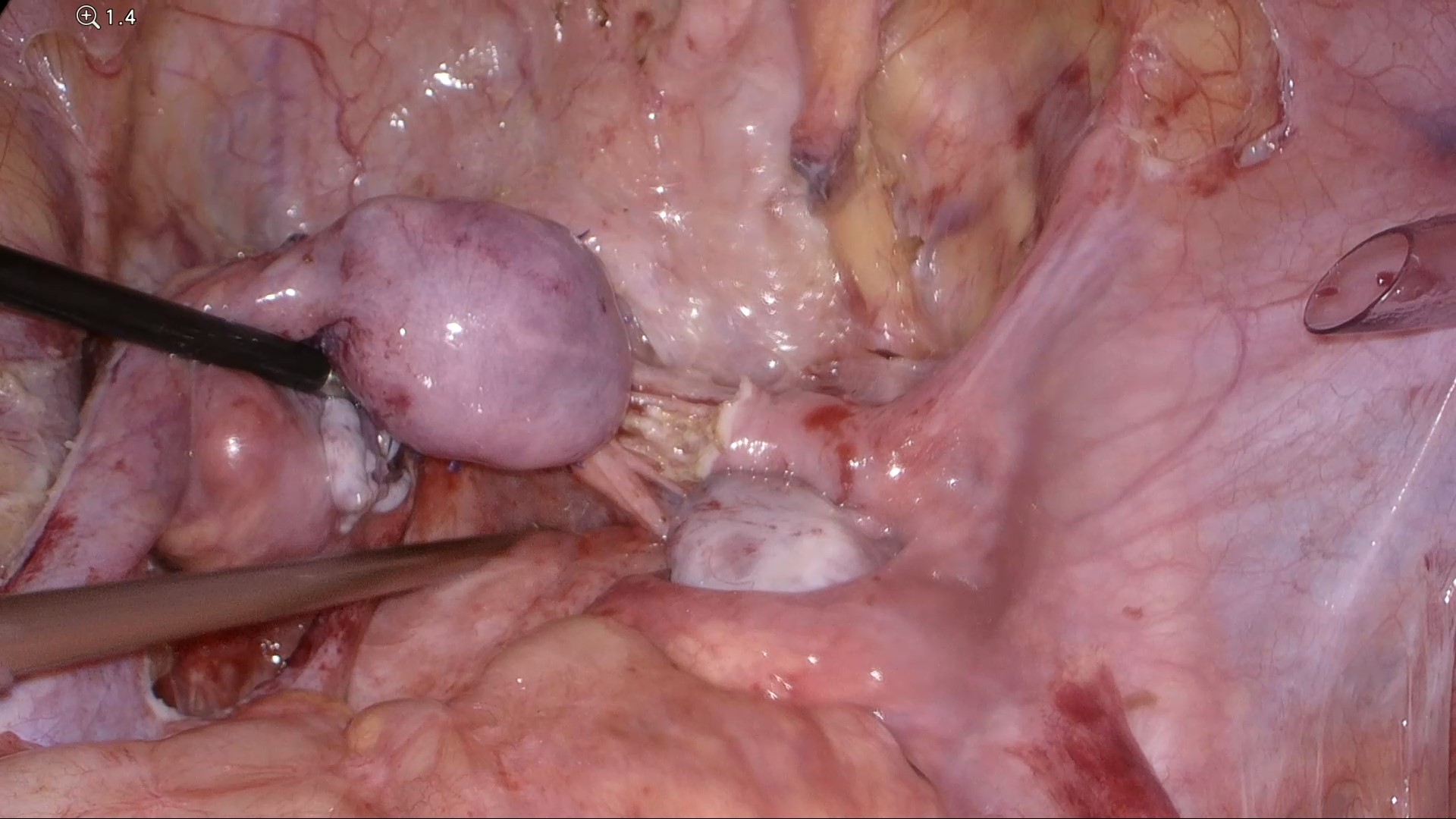


Supplementary figure 8 Anastomosis of lower uterine outlet and artificial vaginal tip
